# Pain Perception Can Be Modulated by Mindfulness Training: A Resting-State fMRI Study

**DOI:** 10.3389/fnhum.2016.00570

**Published:** 2016-11-10

**Authors:** I-Wen Su, Fang-Wei Wu, Keng-Chen Liang, Kai-Yuan Cheng, Sung-Tsang Hsieh, Wei-Zen Sun, Tai-Li Chou

**Affiliations:** ^1^Graduate Institute of Linguistics, National Taiwan UniversityTaipei, Taiwan; ^2^Neurobiology and Cognitive Science Center, National Taiwan UniversityTaipei, Taiwan; ^3^Graduate Institute of Brain and Mind Sciences, National Taiwan UniversityTaipei, Taiwan; ^4^Department of Psychology, National Taiwan UniversityTaipei, Taiwan; ^5^Institute of Philosophy of Mind and Cognition, National Yang-Ming UniversityTaipei, Taiwan; ^6^Department of Neurology, National Taiwan University HospitalTaipei, Taiwan; ^7^Department of Anesthesiology, National Taiwan University HospitalTaipei, Taiwan

**Keywords:** pain, resting-state, connectivity, mindfulness, perspective shift

## Abstract

The multi-dimensional nature of pain renders difficult a holistic understanding of it. The conceptual framework of pain is said to be cognitive-evaluative, in addition to being sensory-discriminative and affective-motivational. To compare participants’ brain-behavior response before and after a 6-week mindfulness-based stress reduction training course on mindfulness in relation to pain modulation, three questionnaires (the Dallas Pain Questionnaire, Short Form McGill Pain Questionnaire-SFMPQ, and Kentucky Inventory of Mindfulness) as well as resting-state functional magnetic resonance imaging were administered to participants, divided into a pain-afflicted group (*N* = 18) and a control group (*N* = 16). Our results showed that the pain-afflicted group experienced significantly less pain after the mindfulness treatment than before, as measured by the SFMPQ. In conjunction, an increased connection from the anterior insular cortex (AIC) to the dorsal anterior midcingulate cortex (daMCC) was observed in the post-training pain-afflicted group and a significant correlation was found between AIC-daMCC connectivity and SFMPQ scores. The results suggest that mindfulness training can modulate the brain network dynamics underlying the subjective experience of pain.

## Introduction

Pain relates to a sensation that hurts. Pain is a very individual experience. Only the person experiencing it can be certain of its existence and even then may have great difficulty describing it with much accuracy. When it comes to measuring one’s pain objectively, it is virtually impossible. The best way to find out how much pain a person is enduring depends at best on a subjective pain report.

### Mindfulness-Based Stress Reduction (MBSR) and Pain Relief

A recent development in the study of pain indicates that mindfulness meditation brings pain relief rather than merely acting as a placebo (Zeidan et al., 2012). Mindfulness skills make it possible for participants to stay not only attentive but also clearly aware of what they are experiencing at the moment, suggesting that mindfulness meditation can be an effective way to reduce pain and deserves further investigation.

Kabat-Zinn’s world-renowned Mindfulness-Based Stress Reduction (MBSR) Clinic, founded in 1979, provides training to participants so that they may develop the capacity to observe their own thoughts and feelings from a detached perspective (McCracken et al., 2013), and thus see themselves as if they were to “video-tape someone else” (Gallagher, 2000), and in turn to view their pain, together with their pain-elicited emotions and judgments, as transient passing events rather than as reflections of the self or reality (McCracken et al., 2013). MBSR’s effect on chronic pain patients has been well established as an outpatient program in behavioral medicine (Kabat-Zinn, 1982). Inspired by such findings among populations of Westerners, we intend to explore in this study functional magnetic resonance imaging (fMRI) changes vis-à-vis behavioral changes in pain-afflicted Chinese subjects after a 6-week MBSR training intervention.

### MBSR: Attention, Empathy, and Perspective Shift

Mindfulness is, according to Kabat-Zinn (2003), the “awareness that emerges through paying attention on purpose, in the present moment, and non-judgmentally to the unfolding of experience moment to moment.” The role of awareness as a vital dimension of consciousness aiding in diverting one’s sensational involvement with pain is not at all a new idea (Allport, 1988; Schmidt, 1994). Since “paying attention on purpose” to the very moment of existence is the basic skill and the ultimate outcome of MBSR training, we will focus mainly on attention in this study, and the word *awareness* will be included only as it pertains to our discussion of attention in this paper.

Mindfulness-based stress reduction training is in fact a means to cognitively “re-construct” participants’ perception of pain, through developing their capacity to observe their own thoughts and feelings from a detached perspective (McCracken et al., 2013). The purpose of such a perspective shift is to place oneself in another’s position, which is empathy, defined as the capacity to understand or feel what another person is experiencing from within the other being’s frame of reference (Bellet and Maloney, 1991). It should be noted that the capacity to understand from another’s frame of reference is a matter of perspective shifting. A speaker usually takes a stance from his own perspective, but to keep up with the true spirit of empathy, he will need to take the other’s perspective in order to understand what the other is experiencing.

### Brain Regions Related to Attention and Empathy

Mindfulness meditation is typically described as non-judgmental attention to experiences in the present moment, as reviewed in Tang et al. (2015). Their review has elucidated neural mechanisms as being an important topic of study in this domain since the complex mental state of mindfulness is supported by alterations in large-scale brain networks. The study of neural mechanisms is a crucial aspect of attempts to understand the mental state of mindfulness and includes analyses of complex networks such as those involved in resting-state connectivity. Also, Tang et al. (2015) considered exhaustively the current state of research on mindfulness meditation and discussed the methodological challenges the field now faces. Taking into account the several shortcomings discussed by Tang et al. (2015) in existing studies, we propose three methodological improvements in the present study. First, a longitudinal approach is designed to minimize pre-training differences that exist in cross-sectional studies. Using two time points allows us to contrast post-training with pre-training to tease apart differences that existed among participants before training. Second, a control group that receives the same mindfulness training as the experimental group is added. The control group enables us to reduce confounds such as practice, memory, or fatigue found in a one-group longitudinal design. Third, resting-state connectivity is utilized to observe changes in coordination among brain regions related to mindfulness training. The correlation of this functional connectivity with questionnaire performance is analyzed to strengthen our arguments by establishing brain-behavior relationships.

As for previous studies on emotional awareness, we are inspired by McRae et al. (2008) in their research of the role of arousal in the relationship between trait emotional awareness and dorsal anterior cingulate cortex (dACC) activity. The relationship between the dACC and emotional awareness is specific to highly arousing emotional stimuli, such as viewing highly arousing pictures. When this is considered in conjunction with the brain areas involved in pain processing, especially the “pain matrix” described by Tracey and Johns (2010), many findings (e.g., Apkarian et al., 2005) indicate that the dorsal anterior midcingulate cortex (daMCC) and the anterior insular cortex (AIC) are highly relevant to attention, with the former (see Bush, 2011; Fan et al., 2014) being involved in top-down attentional control. According to Brooks et al. (2002), paying attention to a pain stimulus results in activation of the AIC, an area of the brain which a later study (Schweinhardt et al., 2006) reports as being related to both acute and chronic pain. Craig (2002) further confirmed that the AIC contains interoceptive representations that substantialize feelings from the body and emotional awareness, especially the pain-related unpleasant ones (Medford and Critchley, 2010).

The AIC has been claimed to be necessary for empathetic pain perception (Gu et al., 2012). Singer et al. (2004) found that empathic feelings for close others experiencing painful stimuli were associated with bilateral activation of the AIC. All these studies have contributed to the present research in their design of experiments aimed at identifying brain regions responsible for attention control, with hopes to see its correlation with emotion regulation and self-awareness, the three core areas mentioned in Tang et al. (2015).

### Our Working Hypotheses

The present study focuses on the change in signal intensity in areas anatomically related to the processing of nociceptive stimuli, as well as areas responsible for attention-related processes, and areas for empathic processes. Pain is a complex, multidimensional and subjective experience, which cannot be fully accounted for by any modality alone. It affects processes that are of a motor-integratory nature, as well as those that are deemed sensory–discriminative, affective–motivational, and cognitive–evaluative (Kupers, 2006). Based on what we have learned from MBSR training, we hypothesize that one may learn to shift one’s subjective experiences via attention practices and we hope to verify such an effect via the participants’ self-reported surveys and the results of the fMRI scans.

To observe changes related to mindfulness training, we use a two-group longitudinal design by taking pre- and post-training measurements with scans and questionnaires for both a pain-afflicted group and a control group. Thus, a 2 (group: low pain, high pain) × 2 (time: pre, post) mixed ANOVA design is used to explore the training effect on fMRI scans and three self-reported surveys, the Dallas Pain Questionnaire (DPQ), the Short Form McGill Pain Questionnaire (SFMPQ) and the Kentucky Inventory of Mindfulness (KIMS). Moreover, resting-state connectivity is also measured to explore changes in dynamic interactions among brain regions associated with mindfulness training. The changes in resting-state functional connectivity (rsFC) measurements are correlated with changes in the questionnaire measurements to elucidate the neural substrates of pain modulation.

Due to the multiple roles played by the AIC in attention, awareness and empathy networks, we hypothesize that the mindfulness training will influence pain perception by strengthening the AIC’s connectivity with the dorsal ACC in meditators (Grant et al., 2011). The relationship between the AIC and the daMCC is crucial because these two regions are both involved in pain processing and in the attention salience network (Seeley et al., 2007), and therefore we hypothesize that the resting-state fMRI will display increased functional connectivity in brain regions associated with pain modulation, especially the AIC-daMCC connection, as a result of MBSR training.

## Materials and Methods

Thirty-four adult participants were recruited in Taiwan for the fMRI experiment. Eighteen participants were selected as the pain-afflicted group, those who both claimed to suffer from moderate or severe pain and scored greater than 1 on the present pain intensity (PPI) index of the standard McGill Pain questionnaire (Melzack, 1975). The remaining sixteen participants selected as the control group scored less than or equal to 1 on the PPI pain index, indicating mild or no pain. These 34 native speakers of Mandarin Chinese (mean age = 38.59, 25 females) were first given an informal interview to ensure that they met the following criteria: (1) right-handedness, (2) normal or corrected-to-normal vision, and (3) without a history of any language deficit or learning disability. After the interview, informed consent was obtained. Our study was approved by the Research Ethics Committee of National Taiwan University before the training and the experiments were administered.

The established model chosen for our mindfulness practices was a 6-week MBSR intervention developed by Kabat-Zinn (1982). The training consisted of six 2.5-h sessions per week and one 8-h non-speaking session in the 4th week. The participants were asked to learn and practice different kinds of mindfulness meditations during the training, including a body scan, sitting meditation, hatha yoga, walking meditation and other informal practices. The body scan was conducted under spoken directions, guiding the participants to progressively move their attention from their toes to head as they observed the physical sensations of different bodily regions. Sitting meditation involved concentration on one’s own breath while remaining open-minded to thoughts, emotions, and other feelings. Hatha yoga contained gentle exercises and body stretching in order to improve the attentive awareness of one’s physical situation in hopes of finding a balance of mind and body. Walking meditation involved walking with intense attention to changes in one’s own gestures and movement. Lastly, the MBSR trainer demonstrates how to make use of the aforementioned methods so that the participants can use them for pain management and in other aspects of their daily life (Shapiro et al., 2007). During the training, the participants were encouraged to focus only on their own breathing, but had to remain aware of different sensations (e.g., sounds and thoughts), accepting the feelings without being responsive to them. All of these techniques were administered in order to encourage the participants to disengage from their personal thoughts and emotions.

In order to compare the differences between pre- and post-training, both the questionnaires and the resting-state fMRI were employed as our major instruments. The three questionnaires were meant to measure the mindfulness skills of the participants, the effects of pain on each individual’s life, and their thoughts about pain, both before and after the training; the resting-state fMRI was used to measure brain functional connectivity before and after the 6-week training.

In order to understand the progression of pain perception and mindfulness skills, two questionnaires, the DPQ and the SFMPQ, were distributed. In addition, the KIMS was used to obtain the participants’ subjective evaluation of their acquired mindfulness skills. All three questionnaires were translated into Mandarin Chinese for the participating subjects.

The DPQ is designed to assess how chronic pain affects different aspects of an individual’s life, including daily activities, work and leisure activities, feelings of anxiety-depression, and social interest (Lawlis et al., 1989). Each of the DPQ’s sixteen sections consists of a single item with a short question asking to what degree pain has adversely affected a particular aspect of life and a corresponding continuous rating scale divided into 5–8 equal sections on which respondents are asked to place a mark indicating the degree from 0 to 100% that expresses their answer. As suggested in Lawlis et al. (1989), the subjective evaluation of pain experience is an important factor in determining how motivated a person is to seek treatment. The self-reported outcome of the DPQ, on the other hand, is conducive to the understanding of chronic pain and, therefore, serves as one of our pain assessment tools.

The SFMPQ, a short version of the McGill pain questionnaire (MPQ) (Melzack, 1975), is commonly used in evaluating how chronic pain influences participants’ sensory, affective, and present feelings. It consists of two subscales with adjectival pain descriptors, including eleven sensory ones and four affective ones. The PPI index of the standard MPQ is also included in the SFMPQ, whose items are presented according to an intensity rating scale ranging from none, mild, moderate to severe, so designed in order to assess the participants’ subjective pain experience (Melzack, 1987). Due to its accessibility and proven validity, the easy-to-follow SFMPQ was chosen as one of our pain assessment tools.

The KIMS is designed to assess whether or not people can exercise mindfulness skills in their daily lives with regard to four facets: observing, describing, acting with awareness, and accepting without judgment (Baer et al., 2004). Mindfulness practices in the KIMS focus on the participants’ abilities to put their feelings, emotion, perceptions and thoughts into words (Bergomi et al., 2013). Based on the “thinking for speaking” hypothesis articulated by Slobin (1987) that one’s language use may shape one’s cognition and may further affect one’s feelings (of pain), we used the KIMS to assess the connection between cognition (one’s comprehension as well as communicative skills) and sensation (one’s feelings).

A 2 (group: low pain, high pain) × 2 (time: pre, post) mixed ANOVA was conducted on the composite score of the DPQ, SFMPQ, and KIMS questionnaire, respectively. These analyses allowed us to assess the behavioral changes related to mindfulness training intervention.

In both groups, resting-state fMRI was used to detect differences in the functional connectivity in brain regions before and after the MBSR training. Such a comparison may help to discover how the training via linguistic instruction assists in regulating a participant’s specific networks that are involved in the cognition of pain. Before fMRI scanning, participants were instructed to lie in the scanner with their head position secured. The head coil was then positioned over the participants’ head. Scanning was conducted on the Bruker 3T S300 BIOSPEC/MEDSPEC MRI scanner, using a quadrature head coil. During the resting-state fMRI scans, participants were instructed to close their eyes and think about nothing, but had to remain awake (Raichle et al., 2001). Each resting scan lasted for 10 min. The data were collected using a gradient-echo planar pulse (EPI) sequence [repetition time (TR) = 3 s, echo time (TE) = 30 ms, 35 slices oriented to the AC-PC line, flip angle = 90°, matrix size = 64 × 64, voxel size = 3.75 × 3.75 × 3.75, slice gap = 0 mm, field of view (FOV) = 24 cm × 24 cm].

Image preprocessing was performed with the Resting-State fMRI Data Analysis Toolkit (REST) version 1.6 (Song et al., 2011) and SPM5 (Statistical Parametric Mapping). The images were corrected for differences in slice-acquisition time to the middle volume and were realigned to the first volume in the scanning session using affine transformations. No participant had more than 3 mm of movement in any plane according to the averages of the realignment parameters. Co-registered images were normalized to the ICBM EPI template, smoothed using a full-width at half-maximum (FWHM) kernel of 10 mm (Chou et al., 2009), detrended, and bandpass-filtered (0.01–0.1 HZ) to reduce non-neuronal contributions to BOLD fluctuations (Zang et al., 2007; Zou et al., 2008). Afterward, low frequency artifacts were removed with a high-pass filter (128-s cutoff period). The nuisance signals of the six head motion parameters, global mean signal, white matter, and cerebro-spinal fluid were regressed out from each voxel’s time course.

The average BOLD signal time course within the seed of the AIC (radius = 8 mm), centered at [-34,16,6] based on a pain study (Tseng et al., 2010), was correlated to every voxel in the brain for each subject using Pearson’s correlation coefficient. The threshold was set to *p* < 0.01 uncorrected at the voxel level. Before group comparisons, the correlation coefficients were converted to *z*-scores using the Fischer r-to-z transformation. These *z*-score images were entered into the statistical analysis. For each participant, the mean signal time course was computed from the seed (i.e., the AIC) and used as a regressor in a voxel-wise rsFC analysis with the functions of REST toolbox 1.6. For each participant, one whole-brain correlation map was obtained from the first-level analysis. Each map was then r-to-z-transformed in order to yield a normal distribution for parametric second-level group analysis. This level comprised a voxel-wise one-sample *t*-test for both the pre-training and post-training, examining whether the correlation coef?cient (*z*-value) indicates positive rsFC (Baur et al., 2013). Signi?cantly correlated voxels were determined at *p* < 0.05 corrected for FDR (false-discovery error rate) at the voxel level with a cluster size greater than or equal to 10 voxels with the use of the daMCC (radius = 10 mm) as our region of interest based on relevant studies (as reviewed in Bush, 2011).

In order to compare the changes in rsFC, we conducted a 2 (group: low pain, high pain) × 2 (time: pre, post) mixed ANOVA on the rsFC magnitudes as represented by the *z*-value. Finally, to explore the brain-behavior relationship, the rsFC magnitude was correlated to the composite score of the DPQ, SFMPQ, and KIMS questionnaire, respectively, with parametric (Pearson) correlation (Baur et al., 2013). All *p*-values were two-tailed. Correlations were assessed with SPSS (version 20).

## Results

Analysis of the scores on the questionnaires to assess the participants’ pain perception and mindfulness skills showed that the participants’ abilities improved after the 6-week training. A 2 (group) × 2 (time) mixed ANOVA on the SFMPQ revealed a main effect of time (*F* = 8.91, *p* < 0.01), a main effect of group (*F* = 14.25, *p* < 0.01), and an interaction of time × group (*F* = 5.28, *p* < 0.05). The *post hoc* comparisons showed that the change in the composite score of the SFMPQ was significant in the pain-afflicted group (*t* = 3.05, *p* < 0.01), but not in the control group (*t* = 0.82 *p* = 0.42). Moreover, the 2 (group) × 2 (time) mixed ANOVA on the DPQ indicated a main effect of time (*F* = 6.45, *p* < 0.05) and a main effect of group (*F* = 6.23, *p* = 0.01). In addition, the 2 (group) × 2 (time) mixed ANOVA on the KIMS showed that there was a main effect of time (*F* = 75.25, *p* < 0.01) and an interaction effect of group x time (*F* = 5.12, *p* < 0.05). Means and standard deviations for the three questionnaires are included in **Table 1**. The results of the 2 (group) × 2 (time) mixed ANOVAs performed on the scores of the three questionnaires are included in **Table 2**.

**Table 1:** Mean and standard deviation (SD) for scores of the Dallas Pain Questionnaire (DPQ), the Short Form McGill Pain Questionnaire (SFMPQ), and the Kentucky Inventory of Mindfulness (KIMS) for pre- and post-training.

		High pain group (*n* = 18)		Low pain group (*n* = 16)	
		Pre	Post	Pre	Post
		Mean (SD)	Mean (SD)	Mean (SD)	Mean (SD)
DPQ	Daily	38.83 (4.63)	29.04 (3.77)	19.73 (4.32)	14.4 (4.26)
	Working/Leisure	39.72 (4.62)	25.72 (3.62)	17.07 (5.74)	13.62 (5.71)
	Anxiety/Depression	39.44 (2.97)	37.96 (4.18)	32.72 (5.41)	30.1 (5.83)
	Social interests	25.28 (4.19)	19.84 (4.16)	12.55 (3.72)	11.39 (4.05)
SFMPQ	Sensation	12.61 (1.4)	7.78 (0.94)	5.59 (1.7)	3.65 (1.64)
	Affective	4.72 (0.56)	2.86 (0.54)	3.06 (0.87)	2.18 (0.68)
	PPI	2.5 (0.17)	1.52 (0.22)	0.41 (0.12)	0.53 (0.23)
KIMS	Observing	34.53 (2.01)	43.34 (1.43)	32.38 (1.82)	36.06 (2.44)
	Describing	21.88 (1.54)	27.93 (1.44)	25.59 (1.43)	29.12 (1.22)
	Acting with awareness	24.87 (1.66)	31.68 (2.05)	27.24 (1.59)	31.06 (1.44)
	Accepting without judgment	17.78 (1.47)	28.06 (1.11)	22.65 (1.7)	29.83 (1.37)

**Table 2:** 2 (group: low pain, high pain) × 2 (time: pre, post) mixed ANOVA on the Dallas Pain Questionnaire (DPQ), the Short Form McGill Pain Questionnaire (SFMPQ), and the Kentucky Inventory of Mindfulness (KIMS).

		*F*	*p*
DPQ	Group	6.727	0.014^∗^
	Time	6.453	0.016^∗^
	Group × time	1.159	0.29
SFMPQ	Group	14.264	0.001^∗^
	Time	8.913	0.005^∗^
	Group × time	5.277	0.028^∗^
KIMS	Group	0.054	0.817
	Time	75.254	<0.001^∗^
	Group × time	5.124	0.031^∗^

*indicates a significant difference.

**FIGURE 1:**
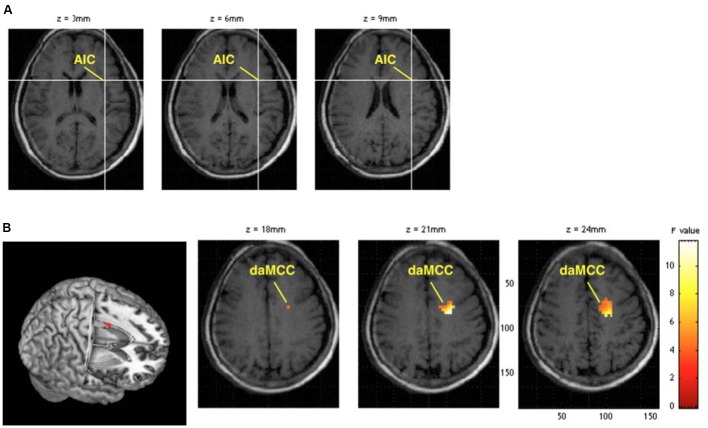
**(A)** The seed region, the anterior insular cortex (AIC). **(B)** The significant increase in functional connectivity of the AIC-dorsal anterior midcingulate cortex (daMCC) after the 6-week training in the pain-afflicted group.

In the fMRI data analysis, a 2 (group) × 2 (time) mixed ANOVA on the rsFC magnitudes revealed an interaction between AIC activity and daMCC activity (the daMCC coordinates [-20,-6,21], *z* = 3.07, *p* = 0.001 uncorrected, **Figure 1**). The correlated voxels were also significant at *p* < 0.05 corrected for familywise errors (FWE), using a sphere of 10 mm radius based on relevant studies (Bush, 2011). The *post hoc* comparisons showed a significant increase in post vs. pre-training rsFC magnitudes in the pain-afflicted group (*t* = 2.68, *p* < 0.01), but not in the control group (*t* = 1.08 *p* = 0.30). Correlation analysis was also conducted between the AIC-daMCC connection strength and the composite score of the DPQ, SFMPQ, and KIMS questionnaire, respectively. The results showed a significant negative correlation between the AIC-daMCC connection strength for the pain-afflicted group, when measured against the composite score of the SFMPQ (*r* = -0.48, *p* < 0.05). There was no significant correlation for the DPQ (*r* = -0.05, *p* = 0.79) or KIMS (*r* = -0.07, *p* = 0.68) questionnaire.

## Discussion

We used a two-group longitudinal design as a methodological improvement over previous studies and found that, according to the SFMPQ questionnaire results, pain was reduced in the pain-afflicted group but not in the control group after MBSR training. Increased connectivity between the AIC and the daMCC was also found in the pain-afflicted group, but not in the control group. Furthermore, a negative correlation was found between the SFMPQ measure and the AIC-daMCC connection strength for the pain-afflicted group. We will center our discussion on how one’s perception of pain may be different as a result of attention training, an MBSR-induced cognitive effect. We will also touch briefly on attention-triggered reappraisal.

### Cognition-Based Attention and Perception of Pain

Based on the results of the questionnaires, significant reduction of pain was observed after the 6-week training course. With the reduction in emotional discomfort and somatic problems, the participants’ quality of life was enhanced, which indicates a positive impact from such attention training.

Clinically, mindfulness-based cognitive training has been found to be effective in regulating the participants’ attention to stressor stimuli, i.e., their pain and the associated self-evaluation of pain, by successfully distracting them from the physical feeling of pain and its related associations using the trained decentering techniques (Garland et al., 2009). For example, to report a stimulus of pain, the participants need to be attentive enough to become alert, to orient themselves toward the source of pain and lastly to detect how they may be affected by it. Decentering allows the participants to become more aware of the experience “from some distance” (McCracken et al., 2013).

During the 6-week training period, we observed that attention plays a pivotal role in shifting our perceptions away from pain, by transforming our associative responses to it, and eventually alleviating it. But, what enables the participants to report the stimuli of pain and the associated evaluation of it?

Our study does hint that one’s application of attention enables a change in one’s neural connections, which leads to the shift of pain sensation, as evidenced by the fMRI scans. By shifting their attention, the participants become detached observers who do not focus on being confronted with pain, but remain in a state of mindfulness; that is, they remain attentive. Their past experience of pain, often associated with negative emotions, is now viewed differently as a result of decentering. This may well expand into a new experience where one learns to be unaffected by obsession with one’s own pain. The natural outcome of such perspective shifting may help reduce pain-related stress, mitigate emotional discomfort, or even yield positive emotions via pain modulation. Thus, by comparing the questionnaires conducted before and after the mindfulness-training course, we observed pain-reduction-induced emotional changes.

### Mindfulness and Cognitive Reappraisal

When it comes to the results of the fMRI imaging, the increased connection strength observed between the AIC and the daMCC can be viewed as a result of the MBSR training in the pain-afflicted group. The AIC plays a pivotal role in deactivating the default mode network (DMN), which consists of brain areas that are activated during mind-wandering or resting states (Menon and Uddin, 2010). The DMN, most notably in the ventral medial prefrontal cortex (vmPFC) and the PCC, is deactivated during meditation—a state requiring constant focus on attention (Brewer et al., 2011). However, the DMN deactivation patterns that occur during meditation may vary from patient to patient (Buckner et al., 2008; Tagliazucchi et al., 2010).

We thus infer that the practice of MBSR may trigger AIC activation, which in turn may inhibit vmPFC activity, a signal reflecting emotional suffering (Apkarian et al., 2011). When receiving nociceptive stimuli, the AIC and the daMCC are both activated, and such co-activation is considered to be responsible for autonomic and emotional processes (Sterzer and Kleinschmidt, 2010). Both the AIC and the daMCC could very well be specific regions of the “salience network” (Sterzer and Kleinschmidt, 2010; Harsay et al., 2012), which “forms the fundamental neuroanatomical basis for all human emotions” (Decety, 2011). This significant difference between the experimental group and the control group indicates that the increased AIC-daMCC connectivity observed could be specifically related to pain perception.

The daMCC is said to take part in cognitive control and emotional regulation (Modinos et al., 2010; Kanske et al., 2011). Also, the daMCC is involved in attentional control (as reviewed in Bush, 2011). Together with the pain matrix proposed by Decety (2011), the strengthened connectivity associated with the participants’ ability to modulate their perception of pain, based on a perspective shift, suggests that this strengthened connectivity is the underlying mechanism for such change in pain perception.

Alternatively, the AIC and the daMCC are often jointly activated, which appears consistent with the idea that both of them serve as complementary limbic sensory and motor regions (Craig, 2009). These two regions work together to form a link between self-recognition and self-control. In the present study, the observed increase in AIC-daMCC connectivity may be related to enhanced self-recognition and self-control due to the MBSR training. The participants’ perception of pain differed after the 6-week training program. We contend that the difference is a result of cognitive re-evaluation made possible via MBSR attention training, in keeping with the contention that a positive shift in perspective is an effective means of coping with stressful events and a good predictor of increased mindfulness, which is well supported by Garland et al. (2009, 2011). In the model advanced by Garland et al. (2011), there exists an upward-spiraling relationship between positive perspective shift and mindfulness: The more mindful one becomes, the more likely one is to use a positive perspective shift as a coping strategy in dealing with previously stressful or painful experiences.

## Conclusion

Our resting-state fMRI results suggest that people can modify their cognition of pain through attention training. The results based on the resting-state fMRIs and questionnaires indicate that a possible mechanism, perspective shift, may be at work during the cognitive regulation of pain perception during the mindfulness training. Regardless, we need to admit that several confounding factors may limit the findings presented so far. One is that we should have investigated the importance of quantity and quality on the effect of MBSR skill training. We also are aware that we should have included a scanning of participants engaged in a cognitive task to further reveal the function and meaning of increased connection strength.

## Future Studies

Although recent studies, especially those involving neuroimaging, have started to identify brain areas and networks that mediate the correlation between mindfulness and attention, the underlying neural mechanisms remain unclear. To gain a better understanding of the neural basis of the changes in the brain, we would like to consider other mechanisms in our future study. For instance, is it via language that pain can be noticed, constructed, and assessed, notwithstanding the fact that it is hard to come by much pain-describing language (Dyer, 2011)? The notion that labeling emotional states can help to regulate negative emotional states is hardly new, as can often be seen in talk therapies which involve individuals instructed to put their feelings into words in hopes of managing or transforming their feelings. Lieberman (2011) further confirms that putting feelings into verbal language activates a region of the brain that is capable of inhibiting various kinds of immediate experience, including affective distress. Perspective-taking is clearly not foreign to language, and empathy (i.e., taking the perspective of someone else) is partially mediated by language (Izard et al., 1988).

## Author Contributions

I-WS, K-CL, K-YC, S-TH, and T-LC designed this study. I-WS, F-WW, W-ZS, and T-LC collected and analyzed data. I-WS, F-WW, K-CL, and T-LC wrote the paper.

## Conflict of Interest Statement

The authors declare that the research was conducted in the absence of any commercial or financial relationships that could be construed as a potential conflict of interest.
